# Cellular and molecular changes and immune response in the intestinal mucosa during *Trichinella spiralis* early infection in rats

**DOI:** 10.1186/s13071-020-04377-8

**Published:** 2020-10-06

**Authors:** María Priscila Saracino, Cecilia Celeste Vila, Melina Cohen, María Virginia Gentilini, Guido Hernán Falduto, Marcela Adriana Calcagno, Estela Roux, Stella Maris Venturiello, Emilio Luis Malchiodi

**Affiliations:** 1 Facultad de Farmacia y Bioquímica, Cátedra de Inmunología-Instituto de Estudios de la Inmunidad Humoral Dr. Ricardo A. Margni (IDEHU), UBA-CONICET, Universidad de Buenos Aires, Buenos Aires, Argentina; 2 Instituto de Investigaciones Cardiológicas, Dr. Taquini, ININCA-UBA-CONICET, Buenos Aires, Argentina

**Keywords:** *Trichinella spiralis*, Gut immunity, Inflammation, Innate immunity cell, Antibodies response, T cell

## Abstract

**Background:**

The main targets of the host’s immune system in *Trichinella spiralis* infection are the adult worms (AW), at the gut level, and the migrant or newborn larvae (NBL), at systemic and pulmonary levels. Most of the studies carried out in the gut mucosa have been performed on the Payer’s patches and/or the mesenteric lymph nodes but not on the lamina propria, therefore, knowledge on the gut immune response against *T. spiralis* remains incomplete.

**Methods:**

This study aimed at characterizing the early mucosal immune response against *T. spiralis*, particularly, the events taking place between 1 and 13 dpi. For this purpose, Wistar rats were orally infected with muscle larvae of *T. spiralis* and the humoral and cellular parameters of the gut immunity were analysed, including the evaluation of the ADCC mechanism exerted by lamina propria cells.

**Results:**

A marked inflammation and structural alteration of the mucosa was found. The changes involved an increase in goblet cells, eosinophils and mast cells, and B and T lymphocytes, initially displaying a Th1 profile, characterised by the secretion of IFN-γ and IL-12, followed by a polarization towards a Th2 profile, with a marked increase in IgE, IgG1, IL-4, IL-5 and IL-13 levels, which occurred once the infection was established. In addition, the helminthotoxic activity of lamina propria cells demonstrated the role of the intestine as a place of migrant larvae destruction, indicating that not all the NBLs released in the gut will be able to reach the muscles.

**Conclusions:**

The characterization of the immune response triggered in the gut mucosa during *T. spiralis* infection showed that not only an effector mechanism is directed toward the AW but also towards the NBL as a cytotoxic activity was observed against NBL exerted by lamina propria cells. 
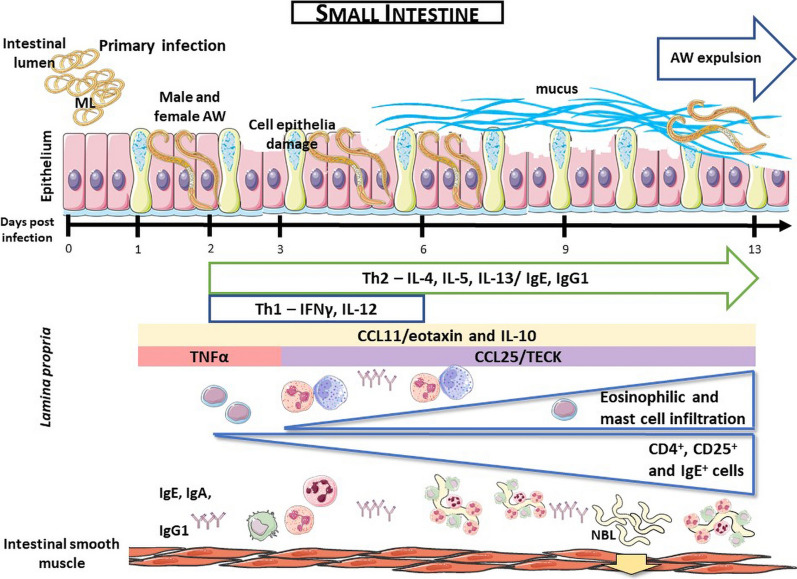

## Background

Parasitic worms infect billions of people annually worldwide and continue to be important human pathogens [1]. *Trichinella spiralis* is a nematode parasite which causes an estimated number of 10,000 cases per year with a mortality rate of about 0.2% [2]. Despite its low mortality rates, the financial cost of this disease is high for both big and small pork meat producers.

Rats play a key role in both the domestic and sylvatic cycles of *T. spiralis*. In the domestic cycle, pigs constitute the main source of infection to humans because they are fed with human food leftovers, which attract rats [3–5]. In the sylvatic cycle, rats are also present and are a food source to wild boars, one of the main *T. spiralis* reservoirs in the wild. Wild boars are hunted and consumed without following any sanitary regulations [4, 6]. As they are natural hosts and since they play a crucial role in both the synanthropic and sylvatic cycles of *T. spiralis*, rats constitute an excellent model to study the disease.

The larval and adult stages of *T. spiralis* are found in the intestinal epithelium, often at the crypt-villus junction, and do not appear to cross the basement membrane. Despite its relatively large size, the worm establishes an intracellular niche, invading several cells simultaneously [7, 8]. This nematode is not sessile in the niche but instead migrates in a sinusoidal pattern, leaving a trail of dead cells behind [9].

A type 2 immune response is essential against gastrointestinal nematodes. Typically, this protective immune response, which causes the expulsion of the adult worm (AW), is T-cell mediated and includes, at the gut level, mastocytosis and goblet cells hyperplasia with secretion of mucus, soluble mediators such as IL-4, IL-5, IL-13, histamine, the production of antibodies (IgE and IgG1) and eosinophilia [10–12]. Despite the existence of effector mechanisms that are common to all nematodes, those accomplishing the parasite clearance from the intestine can be species-specific [11].

The role of IgE in parasitic infections has always been controversial. Some authors suggest that the inflammation produced in the intestinal mucosa during nematode infection, including eosinophilia, is an immunopathological response rather than a protective mechanism [13].

Most research works assessing the intestinal immune response in *T. spiralis* infection have focused on the response generated in Peyer’s patches and mesenteric lymph nodes. It has been demonstrated that the B cell response triggered by other intestinal pathogens is mainly featured by the production of IgA in Peyer’s patches. Conversely, *T. spiralis* has been demonstrated to trigger the secretion of other antibody isotypes [14, 15]. Furthermore, the immune responses elicited in areas such as the lamina propria have been scarcely studied.

Taking into account that the number of lymphocytes present in non-lymphoid regions of the small intestine (lamina propria and epithelium) is higher than that all Peyer’s patches [16], and that the protective activity of IgG and IgE is stronger than that of IgA, the mechanism and dynamics of cells and antibodies responses in non-lymphoid areas should be assessed in *T. spiralis* infection [14, 15]. Accordingly, the aim of this study was to characterize the immune response kinetics in the lamina propria as well as in Peyer’s patches and mesenteric lymph nodes of the small intestine during the intestinal phase of trichinellosis (1–13 days post-infection, dpi). To this end, parameters of type 1 and type 2 immune responses were evaluated, i.e. cytokine and chemokine levels as well as T cells phenotype and the kinetics of secretion of specific antibodies. The capacity of intestinal lamina propria cells to kill the *T. spiralis* NBL was also assessed.

## Methods

### Animals and infection

Two-month-old female Wistar rats or NIH Swiss and N:NIH(S)-nude mice were orally infected through a gastric canula with 2000 or 200 muscle larvae (ML), respectively. ML were obtained from the muscle tissue of Swiss mice by the artificial digestion method [17]. As controls, non-infected animals orally administered with PBS were employed. During all the experiments animals were exposed to 12 h light-dark cycles; room temperature was kept at 21 ± 1 °C and they were provided with water and food *ad libitum*.

### Statistical analysis

The statistical analysis was carried out using the statistical package R [18] and the level of significance was set at 0.05. Data were analysed by one or two-way ANOVA followed by Tukey’s *post-hoc* multiple comparisons test (*nlme* and *multcomp* packages, respectively). In order to test the normality distribution of the data, a graphical analysis was done by a QQ-plot graph and the analysis was done by the Shapiro-Wilk test (*nlme* package). To test homocedasticity, the Levene test was employed (*car* package). When these assumptions were not satisfied by the data sets, alternative analyses were employed, which were detailed in each case. Graphs were generated with GraphPad Prism version 8.0.1 for Windows, GraphPad Software, La Jolla California USA, (www.graphpad.com).

### Parasite stages and antigens

#### ML excretory-secretory products

ML excretory-secretory products (ML-ESP) were prepared according to Nuñez et al. [19]. Briefly, ML recovered from infected rats by the digestion method were washed several times by settling through RPMI supplemented with antibiotics (500 IU/ml penicillin and 500 μg/ml streptomycin; Gibco, Grand Island, NY, USA). Larvae were placed into 75 cm^2^ culture flasks (Corning Costar Corporation, MA, USA) at a concentration of 25,000 ML/ml in RPMI supplemented with antibiotics (100 IU/ml penicillin, 100 μg/ml streptomycin; Gibco) and kept for 18–20 h at 37 °C in 10% CO_2_ in air. ML were then removed, and culture supernatants filtered through a 0.22 μm membrane (Millex-GV, Millipore Co, MA, USA), concentrated 100-fold on a YM-3 membrane (Amicon, Billerica, MA, USA) at 4 °C and dialyzed against PBS. Protein concentration was determined by the BCA protein assay reagent according to manufacturer’s directions (Pierce, Rockford, IL, USA). ML-ESP were kept at -86 °C until use.

#### AW excretory-secretory products

AW excretory-secretory products (AW-ESP) were obtained as described in Nuñez et al. [20]. At necropsy, the upper half of the small intestine of rats infected at 3 dpi was slit open lengthwise and incubated over a double layer of cheesecloth in saline at 37 °C for Baermann separation. After recovery for 2 h, the worms were washed several times by settling through RPMI supplemented with antibiotics (ATB, 500 IU/ml penicillin and 500 μg/ml streptomycin; Gibco) and counted. Worms were placed into 75 cm^2^ culture flasks (Corning) at a concentration of 6000 AW/ml in RPMI supplemented with ATB (100 IU/ml penicillin and 100 μg/ml streptomycin; Gibco) and kept for 24 h at 37 °C in 10% CO_2_ in air. The procedure then continues as for the obtention of ML-ESP.

#### Newborn larvae

Newborn larvae (NBL) were obtained as described previously [21]. Briefly, AW were recovered from the intestine of rats 5–6 days after oral infection with 7000 ML. Worms were cultured in RPMI medium (Gibco) supplemented with ATB (100 IU/ml penicillin and 100 μg/ml streptomycin; Gibco) and 5% foetal calf serum (FCS, Natocor, Villa Carlos Paz, Córdoba, Argentina) at 37 °C in a humidified atmosphere with 5% CO_2_. NBL were collected 2 h later and used immediately. Larvae were alive and in good condition as judged by their motility.

### Intestinal tissue extracts

Intestinal tissue extracts were obtained on 1, 2, 3, 6, 9 and 13 dpi using the PERFEXT method [22] with slight modifications to detect total and specific immunoglobulins (Igs), cytokines and chemokines. Briefly, rats (*n* = 5/timepoint) were bled and infused with PBS plus heparin (10 UI/ml, Sigma-Aldrich, St. Louise, MO, USA) into the heart. The perfused organs were cut into small pieces, placed in an extraction solution containing 90 mM CHAPS (Research Organics, OH, USA) in PBS and protease inhibitors (EDTA-free complete; Roche Diagnostics, Roche Diagnostics, Mannheim, Germany) at 10 µl/mg of tissue and frozen at -86 °C. After thawing, the extraction was performed overnight at 4 °C using a homogenizer. After centrifugation at 12,500× *g* for 10 min at 4 °C, the supernatants were collected, filtered through a 0.22 µm filter (Millipore, Co, MA, USA). These extracts were aliquoted and kept frozen at -86 °C until use.

### Mesenteric lymph nodes, Payer’s patches and lamina propria cells suspensions

Perfused intestines were obtained on 1, 2, 3, 6, 9 and/or 13 dpi as described above. When cell suspensions were obtained from lamina propria, intestines were degreased, and Peyer’s patches were removed. Peyer’s patches were then cut into small pieces and treated with RPMI medium (Gibco) containing EDTA (5 mM; Gibco), dithiothreitol (DTT, 2 mM; Promega, Madison, USA), L-glutamine (1.46 g/100 ml; Gibco), 5% FCS (Natocor) and antibiotics (Gibco) for 30 min to remove the epithelial cell layer. Then, an enzymatic digestion was performed in RPMI medium containing collagenase A (1.5 mg/ml; Roche Diagnostics), DNase (0.1 mg/ml; Roche Diagnostics), L-glutamine (1.46 g/100 ml; Gibco), 5% FCS (Natocor) and ATB (Gibco) for 45 min. Treatments with both solutions were carried out at 37 °C with constant agitation. Subsequently, the cell suspension obtained was filtered through a 20 μm pore nylon mesh. To obtain cell suspensions from Peyer’s patches and mesenteric lymph nodes, each tissue was separated from the intestine and then they were disintegrated over a stainless-steel mesh. The cell suspensions obtained in each case were filtered through a 20 μm pore nylon mesh to eliminate tissue debris.

In all cases, cell suspensions were resuspended and washed twice with PBS plus 5 mM EDTA (Gibco) and 3% FCS (Natocor), and the cells were counted using a hemocytometer and Trypan blue dye (Gibco). The cell viability was invariably higher than 80%. Finally, cell suspensions were suitably resuspended in RPMI medium (Gibco) supplemented with antibiotics (Gibco) and 5% FCS (Natocor).

### Leucocyte formula

Intestinal lamina propria cell suspensions from non-infected rats and rats infected on 3, 6 and 13 dpi were concentrated by cytocentrifugation at 400× *rpm* for 10 min (Shandom Cytospin III, Thermo Fisher Scientific, Waltham, MA, USA) and stained with Giemsa (Merck, Darmstadt, Germany). The leucocyte composition was determined by identifying 200 cells in an optical microscope (Olympus BX51 156; Olympus, Tokyo, Japan).

### Sera and intestinal fluids

From the same rats used to obtain intestinal tissue extracts, sera were collected from blood obtained by cardiac puncture. As a negative control, sera were collected from non-infected rats (non-infected rat sera, NIRS). A serum obtained at 45 dpi, reference cytotoxic serum (RCS), is a pool of sera from three rats infected with 5000 ML/animal presenting anti-NBL surface antibodies (IgE: 512-128; IgG1: 512; IgA: 256-128 and IgG2a: 512-64; antibodies isotypes titers obtained by indirect immunofluorescence (IIF) assay) and whose helminthocytotoxic capacity against NBL was assessed using rat peritoneal leucocytes. When a source of complement was needed, fresh sera from non-infected rats were used.

Intestinal fluids of rats were obtained from white-bled and perfused rats. Briefly, a passage of 5 ml of PBS complemented with protease inhibitors (Roche Diagnostics) was made at 4 °C through the intestinal lumen, immediately centrifuged at 12500×*g* for 10 min at 4 °C to eliminate faecal matter and/or tissue debris. Sera and intestinal fluids were aliquoted and stored at − 20 °C until use.

### Histological and indirect immunofluorescence (IIF) analysis of intestinal tissue

Histological and IIF analysis was performed on formalin fixed paraffin embedded sections obtained from non-infected and infected rats on 1, 2, 3, 6, 9 and/or 13 dpi. Paraffin from tissue sections was removed by gently shaking the slides in 4 consecutive baths of xylene, followed by 2 washes in pre-cooled absolute ethanol, 2 baths of pre-cooled 95% ethanol and 3 baths of saline solution.

For histological evaluation, sections were stained with Giemsa (Biopur, Rosario, Argentina) and hematoxylin-eosin (H&E, Biopur). Specific staining was used for eosinophils (Luna technique) [23], mast cells (Alcian Blue 86Y-Safranin plus counter-stain with 5% yellow methanol) (Holblon and Sohne, Leipzig, Germany) [24], and goblet cells (Alcian Blue 8GX -PAS/Gill’s Hematoxylin) (Cl. 74240; Mallinckrodt, St. Louis, MO, USA and Biopur, respectively). For IIF analysis, cell labelling was performed by incubating tissue sections as previously described [25], except for CCL25/TECK^+^ cells, in which a goat anti-CCL25 (Santa Cruz Biotechnology, Inc., Santa Cruz, CA, USA) followed by FITC-conjugated donkey anti-goat antisera (Sigma-Aldrich) was used.

In all cases, cell types were counted by two independent observers in 15 villus-crypts units (VCU) randomly selected for each sample at a 400× magnification using an epifluorescence microscope (Olympus BX51 156). Results were expressed as the mean of the positive cells/VCU ± standard deviation (SD). Data were modelled by adjusting to the negative binomial distribution followed by Tukey’s *post-hoc* multiple comparisons test (*MASS* and *multcomp* packages, respectively).

### Detection of cytokines and chemokines in intestinal tissue extracts

Commercial ELISA kits were used to determine IL-4, IL-10, IFN-γ, TNF-α (BD Biosciences, San Jose, CA, USA) and CCL11/eotaxin (R&D Systems, Minneapolis, MN, USA) levels in intestinal tissue extracts obtained from infected and non-infected rats. Samples were run in duplicate and cytokine or chemokine concentrations were obtained by extrapolation in calibration curves. Results were expressed in pg/ml or ng/ml ± SD.

The levels of IL-5, IL-12 and IL-13 were determined by an immunoelectrotransfer blot assay as previously described [25]. Membranes were analysed by densitometry using the Image J software (NIH, Bethesda, MD, USA), and the cytokine levels were expressed as mean arbitrary units ± SD and relativized to the α-tubulin band. All reactions were performed in duplicate using ITEs obtained from two independent groups of rats (*n* = 5 at each dpi studied).

### Histamine detection in intestinal fluids

Since the histamine secreted by mast cells has an impact on mucosal immunity, its levels were measured in intestinal fluids at different times pi. The histamine concentration in intestinal fluids obtained from infected (1, 2, 3, 6, 9 and 13 dpi) and non-infected rats was performed by the commercial ELISA kit Histamine (Life Science Format, Neogen, MI, USA). Results were obtained by extrapolation in the calibration curve and expressed in ng/ml ± SD.

### ELISPOT assay

To detect the presence of total and anti-ML-ESP IgA, IgE, IgG1 and IgG2a, mesenteric lymph nodes, Peyer’s patches or lamina propria intestinal secreting cells (Ig SCs), ELISPOT assays were performed on 3, 6 and 13 dpi as previously described [25]. Spots were counted using an Immunospot reader (CTL, Cleveland, OH, USA) and results were expressed as the mean Ig SCs/10^6^ leucocytes ± SD. Reactions were performed in triplicate (*n* = 5/dpi).

### Detection of total and specific Igs in intestinal tissue extracts

The levels of total IgA, IgE, IgG1 and IgG2a were determined by capture ELISA employing a commercial kit (Bethyl Laboratories, Inc., Montgomery, TX, USA) following the manufacturer’s instructions. Concentrations were obtained by processing and extrapolation from calibration curves performed with standard solutions and results were expressed in μg/ml or ng/ml ± SD.

Anti-ML-ESP or AW-ESP IgA, IgE, IgG1 and IgG2a were determined by indirect ELISA as described by Gentillini et al. [25]. Briefly, 96-well flat-bottomed PolySorp polyvinyl microtiter plates (Nunc, Roskilde, Denmark) were coated with antigen at a concentration of 5 μg/ml in carbonate buffer pH 9.6. After overnight (ON) incubation at 4 °C and between each step, plates were washed with PBS plus 0.05% Tween 20 (PBST-0.05%). Plates were blocked employing a 5% bovine seroalbumin (BSA) solution. ITE were added properly diluted in PBST-0.05% and incubated for 1 h at 37 °C and ON at 4 °C, followed with the corresponding anti-immunoglobulin antisera (Bethyl) suitably diluted in PBST-0.05%. After incubating 2 h at 37 °C, reactions were developed employing a tetramethylbencidine (TBM; BD Biosciences)/H_2_O_2_ solution. Colour reactions were read at 450–600 nm and results were expressed as the mean optical density (OD) ± SD.

Anti-NBL IgE, IgA, IgG1 and IgG2a were detected using IIF assay on slides containing methanol-fixed NBL. All reaction steps were carried out at 37 °C. Slides were incubated 30 min with 15 μl of ITE samples suitably diluted in PBS with 0.1% Tween (PBST-0.1%) and followed by the addition of 15 μl of a goat anti-rat IgE, a goat anti-rat IgA, a goat anti-rat IgG1 or a goat anti-rat IgG2a (Bethyl Laboratories) and then followed with a FITC-conjugated anti-goat IgG serum (Sigma-Aldrich) suitably diluted in PBST-0.1% plus Evans blue. Between each step, slides were washed 3 times with PBST-0.1%. Slides were then air-dried and mounted using buffered glycerin. ITE samples were considered positive when fluorescence of parasite surface was observed with an epilumination microscope (Olympus). Positive and negative controls were included in each assay. Anti-*T. spiralis* immune serum and uninfected rat serum and ITE were employed as positive and negative controls, respectively. When possible, some ITE samples were titered. Experiments were performed in duplicate.

### *Ex vivo* ADCC assay

*Ex vivo* antibody-dependent cellular cytotoxicity (ADCC) assays were designed in order to evaluate the biological activity of intestinal lamina propria cells against NBL. Intestinal lamina propria cell suspensions were obtained from infected rats on 3, 6 and 13 dpi and from non-infected rats as described in “Obtention of mesenteric lymph nodes, Payer’s patches and lamina propria cells suspensions”. For the ADCC 25 μl of a suspension containing approximately 50 live NBL in RPMI medium (Gibco) supplemented with ATB (Gibco) and 5% FCS (Natocor) were added to 8-well flat-bottomed strips (Nunc). Then, 20 μl of either SCR or NIRS, 5 μl of fresh serum as source of complement and 100 μl of intestinal lamina propria cell suspensions (the leucocyte/NBL ratio used was 6000/1) were added.

The NBL mortality percentage (M%) was calculated according to the following formula: M% = [(NBL_i_ − NBL_f_)/NBL_i_] × 100, where NBL_i_ and NBL_f_ are the numbers of living NBL, judged by their motility, counted at the beginning and at the end of the reaction respectively. Results were expressed as the mean of mortality percentages of NBL ± SD obtained from five independent experiments performed in duplicate.

## Results

### Histological changes in the intestinal tissue during early *T. spiralis* infection

**Fig. 1:**
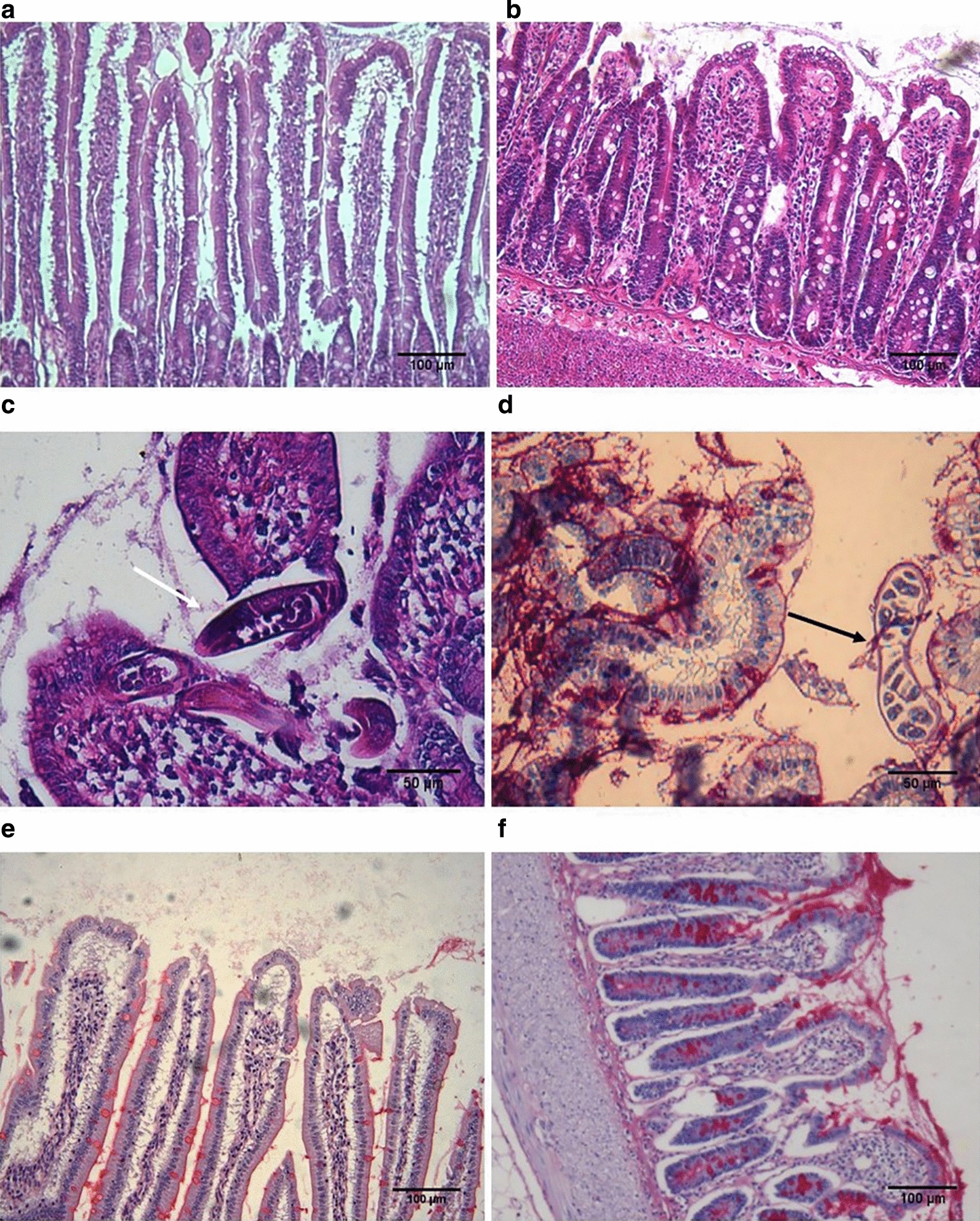
Histopathological changes in the intestine during the early phase of infection by *T. spiralis*. Hematoxylin and eosin staining (**a**, **b**, **e**) and Hematoxylin/Alcyan Blue-PAS staining (**c**, **d**). Sections from non-infected (**a**) and infected rat small intestine showing villi atrophy (**b**), goblet cell hyperplasia and mucus hypersecretion (**d**) surrounding the parasite in the intestinal lumen (**c**, white arrow) and intestinal mucosae damage caused by the AW (**d**, black arrow). **a**–**d** original magnification 10×; **e** and **f** original magnification 20×; **a** is normal rat intestine while **b**–**f** are from rat intestine on 6 dpi. *Scale-bars*: **a**, **b**, **e**, **f**, 100 µm; **c**, **d**, 50 µm

The histological evaluation of intestinal tissue sections from rats infected with *T. spiralis* stained with Giemsa and hematoxylin-eosin showed histological characteristics corresponding to structural alterations of the intestinal mucosa from 4 dpi in 100% of the analysed animals. An intense intestinal inflammation was observed throughout the studied period with a shortening of the intestinal villi (Fig. 1b), as compared to samples from uninfected animals (Fig. 1a) and destruction of the epithelium caused by the direct action of the parasite (Fig. 1c, d). Likewise, goblet cell hyperplasia with increased mucus secretion was observed (Fig. 1e, f) surrounding the parasite in the intestinal lumen, which may be acting as an unspecific mechanism against AW (Fig. 1d).

**Fig. 2:**
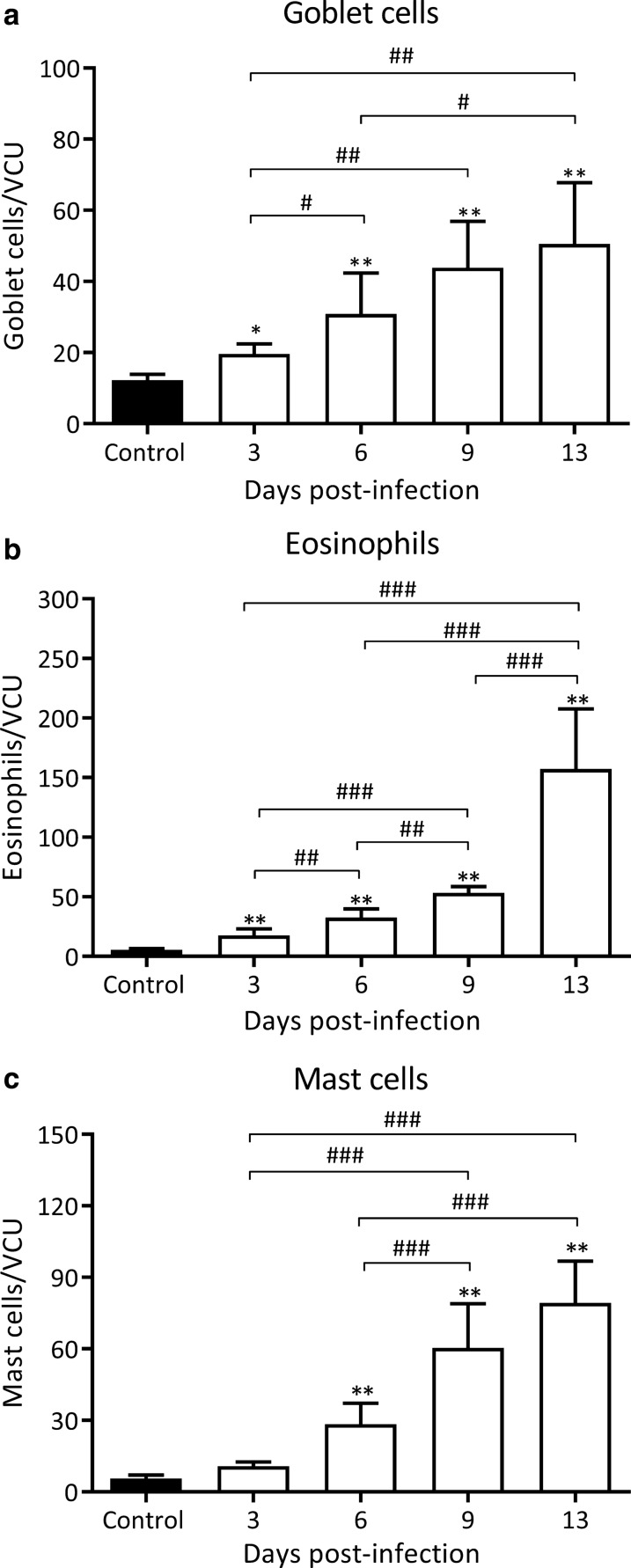
Kinetics of the appearance of innate effector cells in the small intestine during early *T. spiralis* infection. Goblet cells (**a**) were counted in the intestine epithelium using Alcian blue-PAS/Gill’s hematoxylin staining while eosinophils (**b**) and mast cells (**c**) were counted in the intestinal lamina propria using the Luna method and Alcian blue/saphranine stain, respectively. Cells were counted in non-infected and different dpi in tissue sections at 400× magnification per 15 villous crypt units (VCU) by two independent observers and results are expressed as mean of cell numbers/VCU ± SD from 5 rats/dpi. Data were analysed by adjusting to the negative binomial distribution followed by the Tukey’s multiple comparisons test (*MASS* and *multcomp* packages, respectively). Asterisks (*) indicate significant differences between infected and non-infected animals: *P < 0.05, **P < 0.001. Numerals (#) above bars indicate significant differences between days: ^#^*P* < 0.05, ^##^*P* < 0.01; ^###^*P* < 0.001

Eosinophil, mast cell and goblet cell counts were determined in infected rats on 3, 6, 9 and 13 dpi in intestinal tissue sections stained with the Luna technique, Alcian Blue/safranine and Alcian Blue-PAS/hematoxylin, respectively (Fig. 2). Goblet cell counts rose significantly as early as 3 dpi reaching values that were 4-fold higher as compared to non-infected animals on 13 dpi (50.5 ± 17.28 *vs* 12.27 ± 1.5 goblet cells/VCU, *Z*_13 dpi *vs* control_ = 8.83, *P* < 0.001; Fig. 2a). As shown in Fig. 2b, the number of eosinophils also increased progressively until 13 dpi, a timepoint at which a marked increase was observed, reaching values that were 28-fold higher than those observed in non-infected animals (157.4 ± 50.1 *vs* 5.5 ± 1.05 eosinophils/VCU, *Z*_13 dpi *vs* control_ = 16.11, *P* < 0.001; Fig. 2b). The mastocyte infiltrate also increased from 3 dpi until 13 dpi, a timepoint at which a 14-fold increase was observed, as compared to control animals (79.3 ± 17.56 *vs* 5.5 ± 1.5 mast cells/VCU, *Z*_13 dpi *vs* control_ = 14.52, *P* < 0.001; Fig. 2c).

**Fig. 3:**
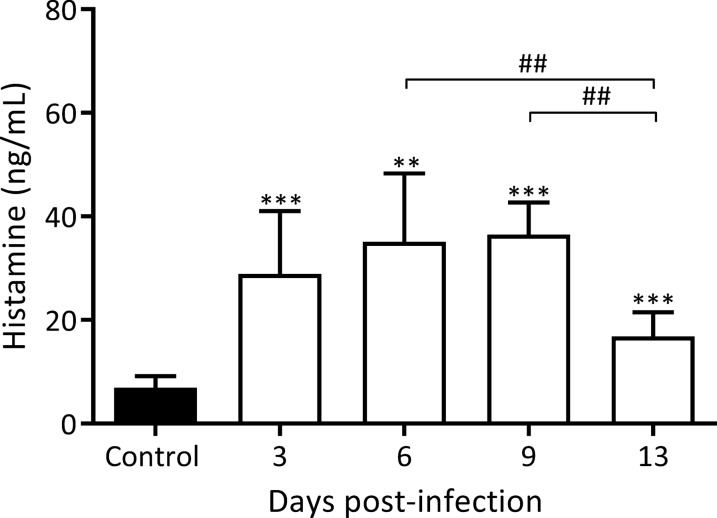
Kinetics of histamine in intestinal fluids from small intestine during early *T. spiralis* infection. Histamine concentrations were determined using a commercial ELISA kit. Results are expressed as the concentration mean ± SD from 5 rats/dpi and 5 control rats. Data were analysed by one-way ANOVA followed by Tukey’s multiple comparisons test (*nlme* and *multcomp* packages, respectively; α = 0.05). Asterisks (*) represent significant differences between infected and non-infected animals: *P < 0.05, **P < 0.01, ***P < 0.001. Bars indicate significant differences among days. Numerals (#) above bars indicate significant differences between days: ^##^*P* < 0.01

Histamine levels in rat intestinal fluids were also quantified on 3, 6, 9 and 13 dpi and the results are shown in Fig. 3. Significantly, the appearance of mast cells in the intestinal lamina propria was accompanied by a histamine release, with an significant increase in histamine levels detected from 3 dpi on, and reaching a maximum concentration on 9 dpi, that is, a 5.2-fold increase with respect to baseline (36.5 ± 2.5 *vs* 6.9 ± 0.7 ng/ml, 9 dpi and control, respectively, *F*_(6, 34)_ = 27.10, *P* < 0.0001; *Z*_9 dpi *vs* control_ = 11.11, *P* < 0.001; Fig. 3), and decreasing on 13 dpi.

### Adaptive immunity in the small intestine

During the enteral phase of the infection with *T. spiralis*, we studied the humoral response, mainly as regards the appearance, kinetics and location of different Igs isotypes as well as the presence of different adaptive immunity cells at lamina propria. Thus, CD4^+^, CD8^+^ and the TCR^+^ cell counts were determined as well as the number of IgA^+^ and IgE^+^ cells present in the small intestine. The anti-*T. spiralis*-ESP IgE, IgA, IgG1 and IgG2a were also determined in cells from intestinal Peyer’s patches, mesenteric lymph nodes and lamina propria.

#### T cell phenotype and kinetics of IgA^+^ and IgE^+^ cells in the small intestine

The cellular phenotype of cells from at lamina propria obtained by evaluating the tissue sections by IIF are presented in Table 1. From 2 dpi on, CD4^+^ T cells increased progressively until the end of the period studied, a timepoint at which, counts reached values that were 3.5-fold higher that those observed in control animals. From 2 dpi on a significant and progressive increase in CD25^+^ cell counts were also observed until the end of the period studied.

**Table 1:** Cell phenotype in the intestinal lamina propria during the early phase of *T. spiralis* infection

	No. of positive cells/VCU					
	CD4	CD25	CD8α	CD8β	TCRαβ	TCRγδ
Control	75.2 ± 12.5^a,^ ^c^	61.6 ± 5.9^c^	162.4 ± 23.5^c^	129.8 ± 26.1^c,^ ^e,^ ^g^	177.2 ± 39.4^c^	151.4 ± 20.7^c^
2 dpi	95.2 ± 2.3^b^	90.2 ± 8.2^d^	215.2 ± 55.5	254.6 ± 29.4^d^	223.8 ± 63.9^d^	241.4 ± 63.9^d^
4 dpi	161.4 ± 18.5^d^	153.0 ± 18.4^d^	215.0 ± 29.7^d^	176.2 ± 12.9^f^	236.6 ± 32.1^d^	208.2 ± 16.4^d^
6 dpi	185.0 ± 20.8^d^	150.4 ± 6.5^d^	87.0 ± 37.8^d^	87.2 ± 25.5^d^	79.4 ± 6.3^d^	79.2 ± 9.9^d^
13 dpi	263.0 ± 36.8^d^	175.0 ± 12.9^d^	193.0 ± 24.8	183.2 ± 21.1^h^	141.2 ± 12.3	155.4 ± 5.9

*Notes*: Results are expressed as mean of number (no.) of positive cells/VCU ± SD (*n* = 5/dpi and *n* = 5, control) at a 400× magnification. Data were analysed by the negative binomial distribution followed by Tukey’s multiple comparisons test (*MASS* and *multcomp* packages, respectively). Significant differences between infected and control rats: *P* < 0.05: a *vs* b; *P* < 0.001: d *vs* c; *P* = 0.008: e *vs* f; *P* = 0.014: g *vs* h

The numbers of CD8α^+^ cells increased significantly only on 4 dpi and decreased significantly to almost half of the control values on 6 dpi and returning to control values on 13 dpi. CD8β^+^ cells followed a similar kinetics with significant increases on 2 and 4 dpi. On 6 dpi, CD8β^+^ cells decreased to almost half of the control values but increasing significantly again on 13 dpi.

**Fig. 4:**
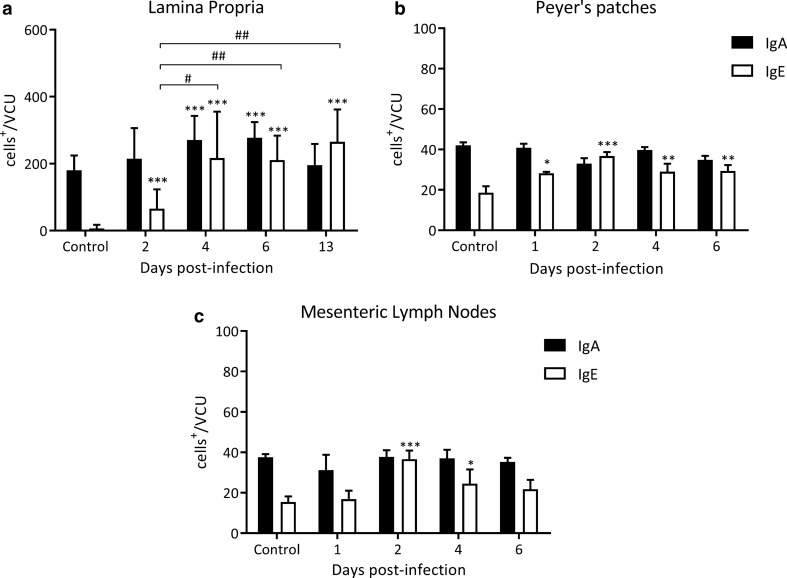
Kinetics of the appearance of IgA^+^ and IgE^+^ cells in the small intestine during early *T. spiralis* infection. Cells were counted at different dpi in tissue sections at 1000× magnification per 15 villous crypt units (VCU) by two independent observers. Results are expressed as numbers of positive cells mean/VCU ± SD from 5 rats/dpi and 5 control rats. Data were analysed by adjusting to the negative binomial distribution followed by the Tukey’s multiple comparisons test (*MASS* and *multcomp* packages, respectively). Asterisks (*) indicate significant differences between infected and non-infected animals: *P < 0.05, **P < 0.001. Numerals (#) above bars indicate significant differences between days: ^#^*P* < 0.05, ^##^*P* < 0.01

TCRαβ^+^ cell counts were found to be significantly elevated from 2 to 6 dpi, when they decreased drastically below control values, to increase again towards 13 dpi to control values. TCRγδ^+^ cell counts showed a similar kinetics, increasing significantly to reach values that were 1.5-fold higher, as compared to control animals on 2 and 4 dpi, then decreasing to almost half of the control values on 6 dpi, and finally returning to control values on 13 dpi. The lamina propria IgA^+^ cell counts showed statistically significant differences between 4 and 6 dpi (270.6 ± 32.2, 277.3 ± 21.1 *vs* 180.0 ± 19.7 IgA^+^ cells/VCU 4 and 6 dpi *vs* control, respectively, *Z*_4 dpi *vs* control_ = 4.55, *P* < 0.001 and *Z*_6 dpi *vs* control_ = 5.17, *P* < 0.001; Fig. 4a) decreasing to control values on 13 dpi. No significant changes were found in cell counts in neither the Peyer’s patches nor the mesenteric lymph nodes, as compared to control animals in any of the days studied (Fig. 4b, c).

In parallel, a significant increase of IgE^+^ cell counts were observed in the lamina propria from day 2 to 13 dpi, a timepoint at which values reached a 40-fold increase with respect to control values (Fig. 4a). In Peyer’s patches, an increase was found from 1 dpi until the end of the period studied, although maximum values were observed on 2 dpi (36.8 ± 0.8 *vs* 18.6 ± 1.4 IgE^+^ cells/VCU, 2 dpi and control, respectively, *Z*_2 dpi *vs* control_ = 5.36, *P* < 0.001; Fig. 4b). Finally, a maximum twofold increase in IgE^+^ cell counts was observed on 2 dpi in mesenteric lymph nodes (36.6 ± 1.9 *vs* 15.4 ± 1.2, IgE^+^ cells/VCU, 2 dpi and control, respectively, *Z*_2 dpi *vs* control_ = 6.36, *P* < 0.001; Fig. 4c).

#### Presence of Ig SCs against ML-ESP at intestinal Peyer’s patches, mesenteric lymph nodes and lamina propria

**Fig. 5:**
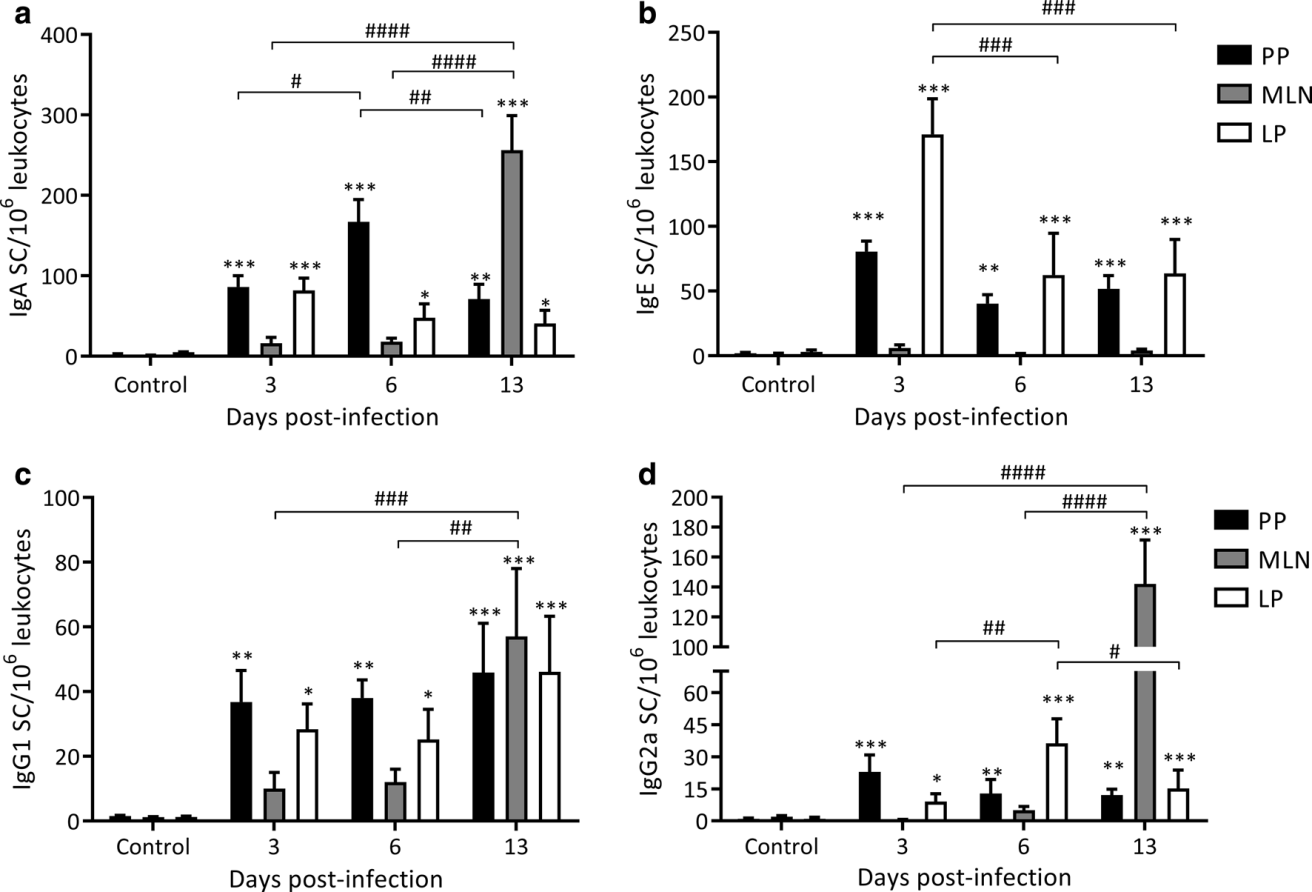
Kinetics of specific Igs secreting cells (Ig SCs) in the small intestine during early *T. spiralis* infection. Ig SCs were determined by ELISPOT assay in Payer’s patches (PP), mesenteric lymph nodes (MLN) and lamina propria (LP). Results are expressed as Ig SCs/10^6^ leucocytes mean ± SD obtained from 3 independent experiments performed with groups of 5 rats/dpi and 5 control rats. Data were analyzed by two-way ANOVA followed by the Tukey’s multiple comparisons test (*nlme* and *multcomp* packages, respectively). Asterisks (*) indicate significant differences between infected and non-infected animals: * *P* < 0.05, **P < 0.01, *** *P* < 0.001. Numerals (#) above bars indicate significant differences between days: ^#^*P* < 0.05, ^##^*P* < 0.01, ^###^*P* < 0.001, ^####^*P* < 0.0001

To determine the sequence of secretion of anti-ML-ESP Igs occuring during the enteral phase of infection with *T. spiralis*, the presence of IgE, IgA, IgG1 and IgG2a anti-ML-ESP secretory cells in Peyer’s patches, mesenteric lymph nodes and lamina propria cell suspensions from infected and non-infected rats were studied using ELISPOT. Results are depicted in Fig. 5. Specific Ig SCs of the four isotypes were detected on 3, 6 and 13 dpi in Peyer’s patches and lamina propria. The highest numbers of IgE SCs were found in the lamina propria on 3 dpi, with values that were 57-fold higher than those of controls (171.1 ± 27.6 *vs* 3.0 ± 1.5 IgE SCs/10^6^ leucocytes, 3 dpi *vs* control, respectively, *F*_(3, 204)_ = 48.21, *P* < 0.0001; *Z*_3 dpi *vs* control_ = 19.43, *P* < 0.0001; Fig. 5b) and, remarkably, even higher than the numbers of IgA SCs (81.8 ± 15.3 *vs* 5.00 ± 0.48 IgA SCs/10^6^ leucocytes, 3 dpi *vs* control, respectively, *F*_(3, 204)_ = 47.92, *P* < 0.0001; *Z*_3 dpi *vs* control_ = 6.44, *P* < 0.0001; Fig. 5a). In Peyer’s patches and the lamina propria, IgG1 and IgG2a SCs showed a 30-fold increase on 3 dpi, although for IgG2a in the lamina propria, counts decreased from 6 to 13 dpi (Fig. 5c, d, respectively). In mesenteric lymph nodes, IgA, IgG1 and IgG2a SCs increased only on 13 dpi to reach values that were 200-fold higher than those of controls (IgA SCs 256 ± 43.1 *vs* 1.30 ± 0.27 IgA SCs/10^6^ leucocytes, 13 dpi *vs* control, respectively, *F*_(3, 204)_ = 47.92, *P* < 0.0001; *Z*_13 dpi *vs* control_ = 20.16, *P* < 0.0001; Fig. 5a); however, IgE SCs did not increase on any dpi evaluated (Fig. 5b). Taking these data together, it can be speculated that after infection, an early and fast immune response is triggered not only through the traditional activation route, starting in the Payer’s patches, but also in the lamina propria.

#### Production and secretion of total and/or specific Ig isotypes against ML and AW stages

**Fig. 6:**
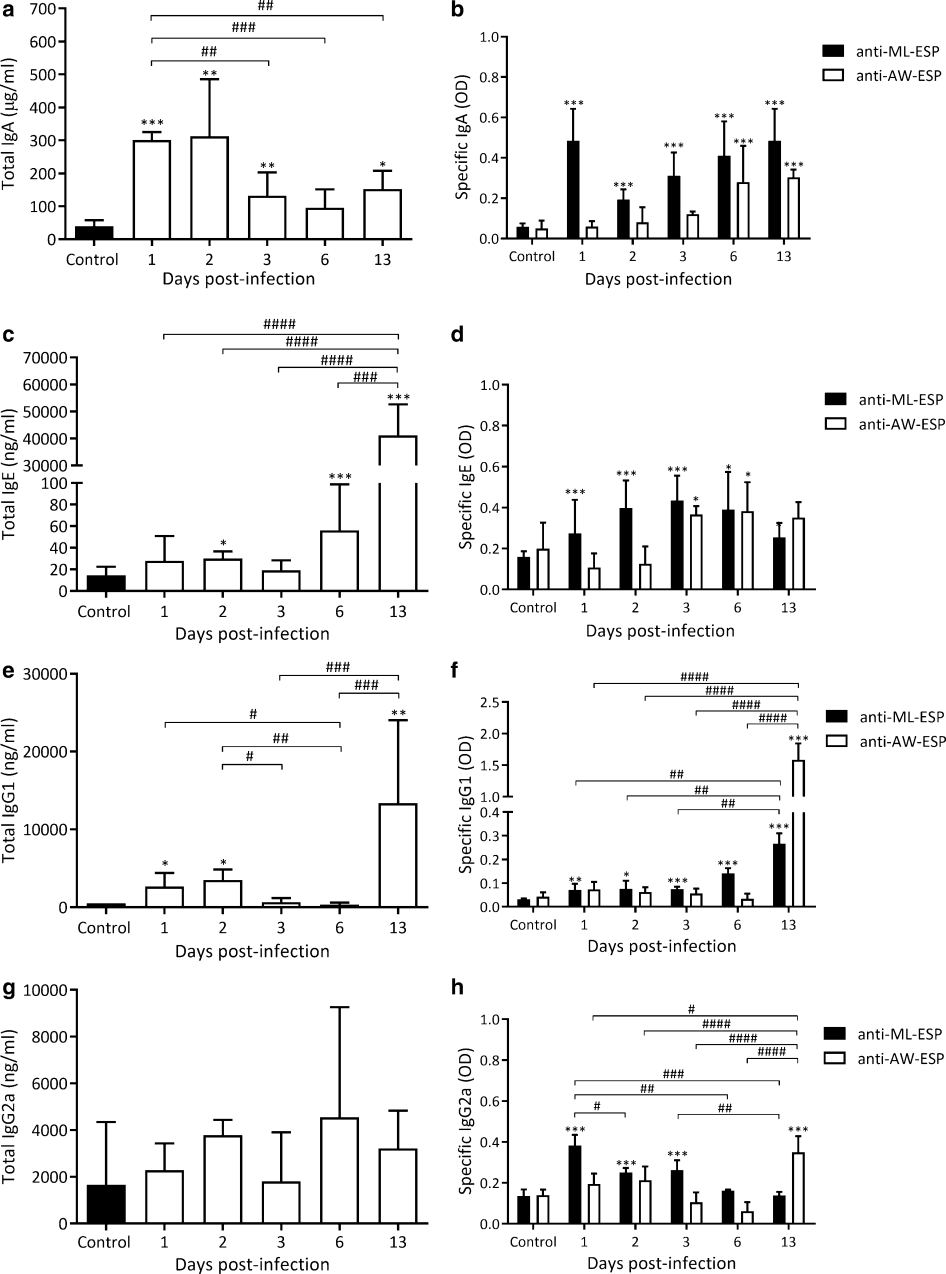
Detection of total and specific Ig isotypes in intestinal tissue extracts during the early phase of *T. spiralis* infection. Total Ig concentrations were determined by commercial ELISA kits whereas levels of specific Igs were determined by an in-house ELISA. Results were expressed as concentration or OD mean ± SD. Data were analysed by one-way ANOVA followed by the Tukey’s multiple comparisons test (*nlme* and *multcomp* packages, respectively; α = 0.05). Asterisks (*) represent significant differences between infected and control animals, *P < 0.05, **P < 0.01, ***P < 0.0001. Numerals (#) above bars indicate significant differences between days: ^#^*P* < 0.05, ^##^*P* < 0.01, ^###^*P* < 0.001, ^####^*P* < 0.0001

In intestinal tissue extracts, total IgA was found to be elevated from 1 to 3 dpi and on 13 dpi. Maximum values were reached on 1 and 2 dpi, showing a 26-fold increase (303 ± 13 and 313 ± 77 *vs* 12 ± 4 μg/ml, *F*_(5, 25)_ = 88.04, *P* < 0.0001; *Z*_1 dpi *vs* control_ = 20.77, *P* < 0.001 and *Z*_2 dpi *vs* control_ = 3.529, *P* < 0.05; Fig. 6a). Total IgE values showed a significant increase on 2 dpi and then decreased on 3 dpi to increase on 6 dpi and reach maximum values on 13 dpi (41,163 ± 5147 *vs* 18 ± 9 ng/ml, *F*_(5, 23)_ = 11.14, *P* < 0.0001; *Z*_13 dpi *vs* control_ = 6.25, *P* < 0.001; Fig. 6c), reaching a 2200-fold increase with respect to control values. Total IgG1 levels were significantly elevated on 1 and 2 dpi, returning to control values on 3 and 6 dpi to increase again (27-fold) by 13 dpi (13,363 ± 5333 *vs* 503 ± 77 ng/ml, *F*_(5, 18)_ = 7, *P* < 0.0001; *Z*_13 dpi *vs* control_ = 4.42, *P* < 0.001; Fig. 6e). Finally, total IgG2a levels were not significantly different from those of control animals throughout the period studied (Fig. 6g).

As for the ML stage, antibodies against ML-ESP were detected during the entire period studied: an 8-fold increase in IgA levels was detected from 1 dpi (0.484 ± 0.064 *vs* 0.059 ± 0.007 OD 1 dpi and control, respectively, *F*_(5, 30)_ = 33.85, *P* < 0.0001; *Z*_1 dpi *vs* control_ = 8.91, *P* < 0.001; Fig. 6b) remaining stable until 13 dpi. IgE was present from 2 dpi, reaching maximum values on 3 dpi (0.434 ± 0.049 *vs* 0.159 ± 0.011 OD 3 dpi and control, respectively, *F*_(5, 30)_ = 14.54, *P* < 0.0001; *Z*_3 dpi *vs* control_ = 5.42, *P* < 0.001; Fig. 6d). IgG1 was detected throughout the evaluated period, with the highest value being observed on 13 dpi, a timepoint in which an eight-fold increase was detected (0.266 ± 0.017 *vs* 0.032 ± 0.001 OD 13 dpi and control, respectively, *F*_(5, 30)_ = 80.73, *P* < 0.0001; *Z*_13 dpi *vs* control_ = 13.20, *P* < 0.001; Fig. 6f). IgG2a levels showed a three-fold increase from the beginning (0.383 ± 0.013 *vs* 0.135 ± 0.021 OD, 1 dpi and control, respectively, *F*_(5, 30)_ = 49.29, *P* < 0.0001; *Z*_1 dpi *vs* control_ = 12.77, *P* < 0.001; Fig. 6h), representing the highest increase in the period. The levels of this isotype then decreased to reach control values on 6 dpi.

Anti-AW-ESP antibodies were detected from 3 dpi until the end of the period studied. IgE was the first isotype to increase, showing a twofold increase on 3 dpi (0.366 ± 0.017 *vs* 0.199 ± 0.052 OD 3 dpi and control, respectively, *F*_(5, 30)_ = 10.32, *P* < 0.0001; *Z*_3 dpi *vs* control_ = 3.001, *P* < 0.05; Fig. 6d). Then, specific IgA levels increased significantly on 6 dpi, reaching maximum levels on 13 dpi (0.357 ± 0.035 *vs* 0.054 ± 0.013 OD 13 dpi and control, respectively, *F*_(5, 30)_ = 21.45, *P* < 0.0001; *Z*_13 dpi *vs* control_ = 8.96, *P* < 0.001; Fig. 6b). In contrast, specific IgG1 values rose sharply towards 13 dpi, showing a 37-fold increase (1585 ± 0.106 *vs* 0.042 ± 0.007 OD 13 dpi and control, respectively, *F*_(5, 30)_ = 43.55, *P* < 0.0001; *Z*_13 dpi *vs* control_ = 14.51, *P* < 0.001; Fig. 6f). Likewise IgG1, IgG2a increased slightly but significantly on 13 dpi, reaching a 2.6-increase, as compared to control values (0.360 ± 0.028 *vs* 0.134 ± 0.009 OD 13 dpi and control, respectively, *F*_(5, 30)_ = 19.37, *P* < 0.0001; *Z*_13 dpi *vs* control_ = 6.77, *P* < 0.001; Fig. 6h). Considering these results and the presence of secreting cells as shown in the previous section, it can be inferred that an early activation of Igs secreting cells occurs in the lamina propria, particularly, those directed against the ML-ESP.

#### Detection of specific Igs in intestinal tissue extracts from normal and athymic mice

In the previous section we described the early appearance of specific antibody isotypes, particularly IgE and IgA, and a significant increase of IgE^+^ cells from 2 dpi. Considering these results, we evaluated the kinetics of anti-ML-ESP and anti-AW-ESP immunoglobulins isotypes in Swiss and nude mice having the genetic background of Swiss mice to evaluate whether the rapid appearance of specific antibodies requires the T-B lymphocyte collaboration.

**Fig. 7:**
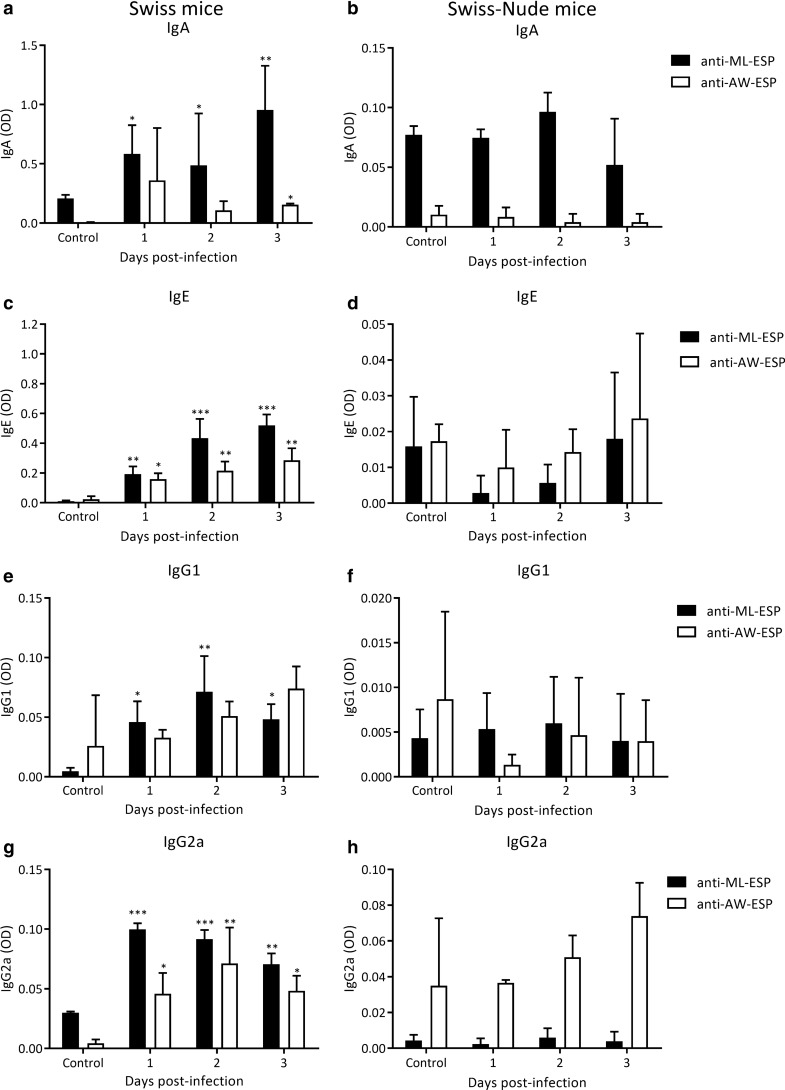
T lymphocytes influence in the kinetics of anti-ML-ESP and AW-ESP Igs in the small intestine during the first days of *T. spiralis* infection. Levels of specific Igs were determined by an in-house ELISA in intestinal tissue extracts from Swiss (**a**, **c**, **e** and **g**) and Swiss-Nude (**b**, **d**, **f** and **h**) mice. Results were expressed as OD mean ± SD from 3 mice/dpi and 3 control mice. Data were analysed by one-way ANOVA followed by the Tukey’s multiple comparisons test (*nlme* and *multcomp* packages, respectively; α = 0.05). Asterisks (*) represent significant differences between infected and control animals, *P < 0.05, **P < 0.01, ***P < 0.001

In Swiss mice, immunoglobulin isotypes showed a similar kinetics to that observed in the rat model: anti-ML-ESP IgA increased from 1 dpi and continued to increase until 3 dpi, while anti-AW-ESP IgA increased significantly from 2 dpi; IgE and IgG2a anti-ML-ESP and anti-AW-ESP increased significantly from 1 and 2 dpi, respectively (Fig. 7). Anti-ML-ESP IgG1 was found to be increased on the three timepoints evaluated, while no anti-AW-ESP response was found (Fig. 7e). In the Swiss-nude model, however, none of the specific isotypes studied increased on 1 dpi (Fig. 7b, d, f, h). It can then be concluded that a T-B lymphocyte collaboration is key for the production and secretion of specific antibodies in *T. spiralis* infection.

#### Evaluation of isotypes anti-NBL surface in sera and intestinal fluids

Igs with specificity for the NBL surface were detected in intestinal fluids with the following pattern: on 6 dpi, 100% of the animals had a positive reaction for IgA, IgE and IgG1 (intestinal fluids titers: 4, 2 and 2, respectively) and while IgG2a was present only in 50% of the animals (intestinal fluids titer: 2); on 13 dpi, 100% of the animals had a positive reaction for IgA, IgE and IgG1 but with higher titers in the case of IgE and IgG1 but lower for IgA (intestinal fluids titers: 16, 8 and 2, respectively). IgG2a were negative in 100% of the animals.

#### Cytokines and chemokines levels in the small intestine

**Fig. 8:**
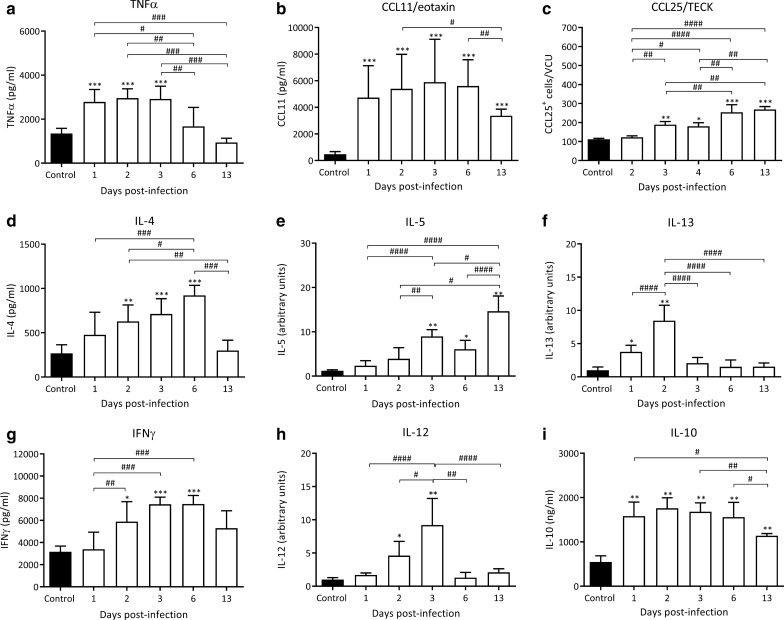
Cytokine and chemokine levels in intestinal tissue extracts during the early phase of *T. spiralis* infection. Concentrations of TNFα, CCL11/eotaxin, IL-4, INFγ and IL-10 in intestinal tissue extracts were determined by commercial ELISA kits whereas CCL25/TECK was determined by immunofluorescence assay. IL-5, IL-13 and IL-12 were determined by an immunoelectrotransfer blot assay. Results are expressed as concentration or number of positive cells/VCU or arbitrary units mean ± SD (*n* = 5/dpi and 5 control animals). Data were analysed by one-way ANOVA except for CCL25/TECK which was modelled by negative binomial distribution followed by Tukey’s multiple comparisons test (*nlme*, *MASS* and *multcomp* packages, respectively; α = 0.05). Asterisks (*) represent significant differences between infected and control animals, *P < 0.05, **P < 0.01, ***P < 0.001. Numerals (#) above bars indicate significant differences between days: ^#^*P* < 0.05, ^##^*P* < 0.01, ^###^*P* < 0.01, ^####^*P* < 0.001

Cytokines and chemokines levels were measured during the enteral phase of the infection (Fig. 8). TNF-α levels were significantly elevated during the first 3 dpi, reaching values that doubled those of control animals (2782.0 ± 566.0; 2958 ± 417.8 and 2919.0 ± 577.3 *vs* 1347.0 ± 235.5 pg/ml; infected rats on 1, 2 and 3 dpi *vs* control rats, respectively, *F*_(5, 27)_ = 15.61, *P* < 0.0001; *Z*_1 dpi *vs* control_ = 4.51, *Z*_2 dpi *vs* control_ = 5.06, *Z*_3 dpi *vs* control_ = 4.94, *P* < 0.001; Fig. 8a). These values returned to basal levels on 6 dpi.

With respect to the chemokines studied, CCL11 showed a significant increase from 1 dpi, with these high levels remaining until 13 dpi, and reaching the maximum value on 3 dpi, which was 12.5-fold higher than control values (5894 ± 3227 *vs* 472.2 ± 197.7 pg/ml, respectively, *F*_(5, 30)_ = 49.64, *P* < 0.0001; *Z*_3 dpi vs control_ = 4.10, *P* < 0.001; Fig. 8b). On the other hand, the levels of CCL25 were significantly higher from 3 dpi on, which continued to rise significantly towards 13 dpi to reach values that were 2.5-fold higher than those of control animals (268.5 ± 15.56 *vs* 111.8 ± 4.5 cells CCL25^+^/VCU, respectively; *Z*_13 dpi vs control_ = 12.14, *P* < 0.001; Fig. 8c).

IL-4, IL-13 and IL-5 are key Th2 cytokines and their kinetics were studied in our model. IL-4 showed a statistically significant increase from 2 to reach a peak value on 6 dpi, reaching approximately a 3.5-fold increase with respect to the control (921.8 ± 113.4 *vs* 267.7 ± 96.9 pg/ml, respectively, *F*_(5, 24)_ = 9.85, *P* < 0.0001; *Z*_6 dpi vs control_ = 6.87, *P* < 0.001; Fig. 8d). After this timepoint, these values descended dramatically towards 13 dpi. IL-5 levels rose from 3 dpi to reach a maximum value on 13 dpi (Fig. 8e). IL-13 levels were elevated only on 1 dpi and 2 dpi returning rapidly to control levels on 3 dpi (Fig. 8f). Taken together the kinetics of cytokines, chemokines, T cell phenotype and IgE SC in the gut mucosa with the appearance of specific IgE antibodies in ITE, these results suggest that the immune response elicited in the small intestine is of a Th2 phenotype.

On the other hand, IFN-γ, a key Th1 cytokine, increased from 2 to 6 dpi to reach values that doubled those of control values (5890.0 ± 1796 and 7473.0 ± 774.5 *vs* 3160.0 ± 511.8 pg/ml, respectively *F*_*(*5, 24)_ = 9.85, *P* < 0.0001; *Z*_2 dpi *vs* control_ = 3.24, *P* < 0.05 and *Z*_6 dpi *vs* control_ = 4.94, *P* < 0.001), decreasing on 13 dpi to control values (Fig. 8g). IL-12 levels rose steadily to reach a peak on 3 dpi (9.20 ± 4.0 *vs* 1.01 ± 0.2 arbitrary units, *F*_(5, 26)_ = 12.13, *P* < 0.0001; *Z*_3dpi *vs* control_ = 5.008 *P* < 0.001, respectively, Fig. 8h) decreasing rapidly to control values on 6 and 13 dpi. IL-10, a cytokine that is related to a regulatory profile, was significantly increased throughout the evaluated period, reaching a maximum 3-fold increase on 2 dpi (1759.0 ± 237.7 *vs* 548.5 ± 136.5 pg/ml, *F*_(5, 24)_ = 20.96, *P* < 0.0001; *Z*_2 dpi *vs* control_ = 2.93 *P* < 0.05; Fig. 8i).

#### Ex-vivo ADCC assay

**Fig. 9:**
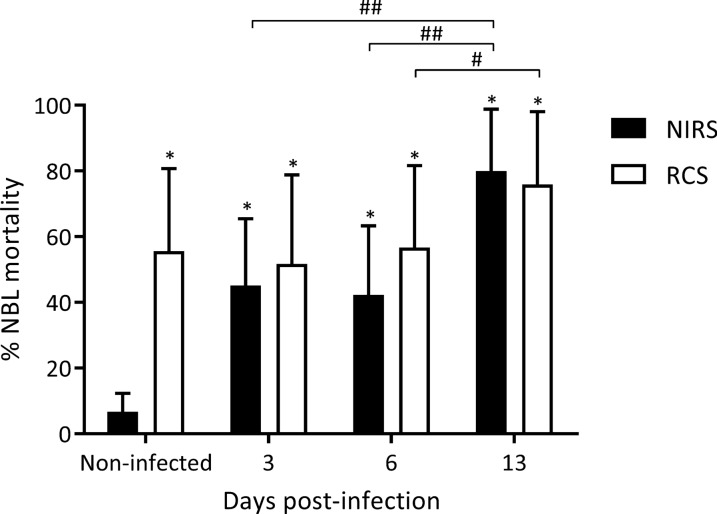
Cytotoxic activity against NBL at the small intestine during early *T. spiralis* infection. Cytotoxic effects of lamina propria cell suspension to NBL suspensions in the presence of reference cytotoxic serum (RCS) or non-infected rat sera (NIRS) were determined by ADCC assays in five independent experiments performed in duplicate (*n* = 5/dpi and 5 control rats). Results are expressed as mean of NBL mortality percentages ± SD. Data were analysed using one-way ANOVA followed by Tukey’s multiple comparisons test (*nlme* and *multcomp* packages, respectively; α = 0.05). Asterisks (*) represent significant differences between infected and control animals: *P < 0.0001. Numerals (#) above bars indicate significant differences between days: ^#^*P* < 0.05, ^##^*P* < 0.01

In ADCC *in vitro* assays, intestinal lamina propria cell suspensions from infected animals were able to kill the parasite with or without the presence of specific antibodies (Fig. 9), with death percentages being higher than those obtained with intestinal lamina propria cell suspensions from non-infected animals. The assays performed in this study revealed that even intestinal lamina propria cell suspensions from 3 dpi were able to kill the NBL in the presence of both RCS or NIRS (Fig. 9). However, on 13 dpi the percentage was the highest (79.92 ± 18.85% and 75.83 ± 22.18% in the presence of both NIRS or RCS, respectively, *F*_(3, 91)_ = 20.11, *P* < 0.0001; *Z*_13 dpi *vs* control_ = 12, *P* < 0.001 and *Z*_13 dpi *vs* control_ = 5.81, *P* < 0.05; Fig. 9).

Interestingly, no significant differences were observed when ADCC experiments were carried out in the presence of serial dilutions of either fresh serum or heat-inactivated serum, indicating that under our experimental conditions, complement factors do not exert cytotoxic effects (data not shown).

#### Analysis of intestinal lamina propria cell suspensions

The composition of intestinal lamina propria cell suspensions used in the *in vitro* cellular cytotoxicity assays was determined by Giemsa staining of cytospin preparations. A change in the cellular composition of these intestinal lamina propria cell suspensions occurred as the infection progressed (Table 2). Intestinal lamina propria cell suspensions from infected animals showed a 12-fold and 8-fold significant increase in the population of neutrophils and eosinophils, respectively. A concomitant decrease in macrophage numbers was found.

**Table 2:** Leucocyte formula of lamina propria cell suspensions from infected and non-infected rats

	Lamina propria cellular suspensions			
	Control	3 dpi	6 dpi	13 dpi
Lymphocytes	52.05 ± 1.9^a^	50.2 ± 2.4	37.2 ± 1.03^c^	45.4 ± 1.8^d^
Macrophages	43.4 ± 2.2^e^	30.1 ± 1.9	32.0 ± 0.8	24.6 ± 5.5^f^
Eosinophils	2.6 ± 0.9^g^	13.2 ± 2.4^h^	24.7 ± 1.9^i^	20.1 ± 1.1^j^
Neutrophils	1.2 ± 0.4^k^	4.5 ± 1.1	5.7 ± 0.4	14.2 ± 1.0^l^

*Notes*: Results are expressed as the mean cell percentage obtained for each leucocyte population in lamina propria cell suspensions from infected rats (*n* = 5/ dpi) and control rats (*n* = 5) ± SD. Data were analysed by two-way ANOVA followed by Tukey’s multiple comparisons test (nlme and multcomp packages, respectively; α = 0.05). Letters represent significant differences between infected and control animals: *P* = 0.0026: c *vs* a; *P* = 0.0053: h *vs* g; *P* = 0.0004: b *vs* a; *P* = 0.0005: d *vs* a, l *vs* k; *P* < 0.0001: c *vs* a, i *vs* g, f *vs* e, j *vs* g

## Discussion

This study was aimed at characterizing the gut immune response in the rat during *T. spiralis* infection. The results presented herein demonstrate that during the enteral phase of infection with *T. spiralis*, between 0 and 13 dpi, a marked inflammatory process occurs, which is featured by the presence of an eosinophilic and mastocytic infiltrate. These results agree with those reported the mouse model [26]. As in the mouse model [26, 27], in rats, *T. spiralis* caused a marked structural alteration of the mucosa characterised by early goblet hyperplasia as well as the presence of mucin in the intestinal lumen to expel the parasite. This activation of goblet cells observed from 1 dpi correlate to the early secretion of IL-13, IFN-γ and IL-4. The role of these cytokines in the induction of goblet cell hyperplasia and mucin secretion has been observed in other helminthiases [16, 28].

The cellular and molecular assessment of the intestinal mucosa indicated that a type 1 oriented response develops from 1 dpi and that this response is characterised by the secretion of with IFN-γ and IL-12. As the infection progresses, the immune response, modulated in part by IL-10, shifts to a mixed Th1/Th2 profile and then to a Th2 profile by 6 dpi with a significant increase of IL-4, IL-5 and IL-13 levels. These results are in line with the immune response developed in the lung mucosa during the enteral phase of *T. spiralis* infection [25]. The gut immune response is characterized by an eosinophilic and mastocytic infiltrate from 1 dpi on and an increase in IgE^+^ and CD4^+^ cells from 3 dpi on. The humoral response was found to be of a Th1 type profile, which shifted to a Th2 phenotype with regulatory components [25, 29].

CCL25 and CCL28 are constitutively expressed by the epithelial cells of the intestine and both chemokines have a key role in directing the migration of T lymphocytes. CCL25, whose levels are regulated by the secretion of TNF-α [30]. Not only do these chemokines mediate lymphocyte traffic to the epithelium (intraepithelial lymphocytes), but also to the lamina propria. Since TNF-α was found to be significantly increased from 1 dpi on, and the levels of CCL25 increased from 3 dpi on, it could be speculated that the increased expression of CCL25 is a consequence of the increase of TNF-α levels (Fig. 8).

Together, the results presented in this work and previous results obtained in our laboratory suggest that there is a simultaneous development of the immune response at the gut and pulmonary mucosa levels. These responses are triggered by a first antigenic stimulation taking place in the gut by the ML stage. This simultaneous mucosal activation might be attributed to cell migration through the MALT [31, 32] and would be dependent on the chemokines (CCL25, CCL28 and CCL11) and cytokines (TNF-α, IL-4, IL-5 and IL-13) released in the gut and the lungs of infected rats (Fig. 8 and [25]).

Mast cells are one of the effector cells involved in the expulsion of AW in mice, although the mechanism has not been elucidated. McDermontt et al. [33] have demonstrated that the kinetics of permeability changes, which are mainly driven by mMCP-I, parallels the kinetics of the adult worm expulsion in mice. Woodbury et al. [34] have found a correlation between RMCP-II serum concentrations in rat and the increase of mast cells counts in the gut villi and the kinetics of AW expulsion. However, the contribution of other soluble effectors in this process cannot be ruled out. Castro et al. [35] have shown an increase in the flow rate of chloride ions when histamine, serotonin, and prostaglandin E2 were externally applied in jejunum sections from *T. spiralis* infected rats. Our results showed that from 3 dpi on, there was a significant increase in histamine levels in the intestinal fluid of infected rats, although the number of mast cells were found to increase significantly from 6 dpi on. Considering these observations and the increase of anti-ESP-ML IgE levels, it could be suggested that there is an activation of the resident mast cells of the intestinal mucosa as early as on 1 dpi. Among the functions of histamine are the contraction of smooth muscle and increase vascular permeability by vasodilation [36], which favours the entry of cell populations recruited to lamina propria and the onset of the AW expulsion. Our results regarding the kinetics of histamine in intestinal fluids are in line with those published by Hegazy [37] who observed a correlation between the histamine content of the intestinal tissues and the kinetics of adult worm expulsion in mice. Moreover, histamine has a dual role as cytokine and chemokine, stimulating the production of bone marrow cells and their recruitment to the release site [37]; in this case, the increase of mast cells and eosinophils in the intestinal lamina propria.

In addition to the presence of mast cells and eosinophils, the inflammatory process taking place in the intestinal mucosa of infected rats from 1 dpi was also found to consist of infiltrating CD4^+^, CD25^+^, CD8β^+^, TCRγδ^+^ and IgE^+^ cells (Table 1, Fig. 4). The significant increase in IgE^+^ and CD4^+^ cells, together with the presence of total and specific IgE and the environment created by the rise of IL-4, IL-5 and IL-13 levels suggest that the CD4^+^ lymphocytes present in the gut would be of the Th2 type.

In helminth infections, TCRγδ^+^ cells are associated, through the secretion of IL-4, IL-5 and IL-13, with the development of a Th2 response [38]. In trichinellosis, TCRγδ^+^ cells would be involved in the stimulation of goblet cells at the intestinal level, since they increase from 4 dpi in the gut epithelia [39]. Considering that TCRγδ^+^ cells also promote epithelial repair [38], it can be postulated that in our model, the role of these cells would be to act as a source of cytokines at the beginning of the infection (coincident with the increase in IL-4 levels, Fig. 8) and promoting, along with M2 macrophages, the repair of the *T. spiralis-*damaged epithelium.

IgA is considered a crucial immunoglobulin in the GALT; however, in this model, an early strong specific IgE response was detected. The anti-ML-ESP IgE kinetics, both secretory cells and Igs present in intestinal tissue extracts, accompanying parasitic stage changes, suggests a protective role of IgE and a polarization of the immune response at the intestinal mucosa towards a Th2 phenotype. This finding is supported by the increased levels of IgG1. High total IgE levels have been reported in helminth infections; however, its biological significance is still unknown. Some authors; however, consider that the induction of the synthesis of high levels of this immunoglobulin would be an evasion mechanism since soluble IgE might bind IgE receptors to prevent specific IgE from binding to the cell surface [40–42]. Watanabe [43] has reported that the increase of total plasma IgE levels in mice is beneficial for the parasite. Unlike *T. spiralis* specific IgE, total IgE levels were not increased on 1 dpi. The increase in anti-ESP-AW IgA levels during the late phase of the intestinal infection could be associated with a reduction of AW fertility and eventually expulsion [44, 45]. On the other hand, the increase in anti-ML-ESP and anti-AW-ESP IgG2a levels correlates with those of IFN-γ on 1 dpi, which would stimulate a type 1 response.

Previous studies conducted in rats infected with *T. spiralis* demonstrated that T cells are activated on 12 dpi in the lamina propria and interact with B cells, which are drained to the mesenteric ganglia and the thoracic duct [46]. In addition, specific IgG and IgE SCs against *T. spiralis* begin to proliferate on 1 and 2 dpi [46]. In the present work, using athymic mice we could demonstrate that the early appearance of all isotypes of Igs is T dependent. These results are in line with those reported by Wang et al. [46]. Considering these findings and the dynamics of secretory cells in the lamina propria, the early presence of anti-ML-ESP Igs (from 1 dpi) would indicate that in *T. spiralis* infection, the entrance of parasite in the intestinal epithelia triggers a rapid and effective immune response in the lamina propria.

It should be noted that the number of activated B cells does not necessarily correlate with the amount of antibody produced. This phenomenon can be evidenced by observing total IgG2a levels, which were not increased at any time, while an increase in the number of IgG2a secretory cells and an increase in anti-ML-ESP IgG2a was observed (Figs. 5, 6, respectively). These observations were also documented by Wang et al. [46] when studying the response of B lymphocytes in the intestine during the infection by *T. spiralis*.

The role of eosinophils in the systemic and pulmonary ADCC mechanism in the defence against *T. spiralis* NBL is known [20, 25, 47, 48]. In the present study, an increase in eosinophils in the lamina propria was demonstrated from 3 dpi on, which is consistent with the increase of CCL11 from 1 dpi in intestinal tissue extracts. These results are in line with those obtained by Gentilini et al. [25] in the lung mucosa but not by those of Venturiello et al. [47] when analysing the phenomenon at a systemic level. These findings would demonstrate that the recruitment of eosinophils at the mucosal level is controlled *in situ* and that this process is independent of the systemic immune system. However, the role of eosinophils, present in the cellular infiltrate of the intestinal lamina propria remains unclear, as it has not been associated with the AW expulsion in a primary infection [26, 49, 50]. Considering the results obtained in ADCC assays, we propose that the function of eosinophils in the lamina propria would be strongly associated with the ADCC mechanism, since intestinal lamina propria cell suspensions from infected animals were found to be helminthotoxic, with the percentages of NBL death increasing together with the eosinophil counts. Since neutrophils and macrophages participate in ADCC mechanism and they were also found in intestinal lamina propria cell suspensions, their role in the death of NBL cannot be ruled out, especially because the neutrophil counts also increase the infection progresses.

Regarding the ADCC mechanism in the intestine and lung mucosa, it is interesting to compare the results presented in this study with those obtained by Falduto et al. [21]. In that work, when lung cell suspensions from non-infected animals were incubated with cytotoxic sera, NBL mortality percentages were lower than the ones found in this study with lamina propria cell suspensions. It is noteworthy that lamina propria cell suspensions from infected animals displayed a cytotoxic activity against NBL even in the absence of cytotoxic sera, while this phenomenon was not observed with lung cell suspensions.

It is also known that eosinophils are involved in antibody-independent cytotoxicity mechanisms during helminth infections [50]. *In vitro* cytotoxicity assays showed that intestinal lamina propria cell suspensions from infected rats were able to attack the NBL through an antibody-independent cytotoxicity mechanism not only on 3 dpi, when we observed an increase in the NBL mortality percentages, but also in the presence of serum anti-NBL antibodies that did not enhance the helminthotoxic capacity (Fig. 9). These results are in line with results previously obtained in our laboratory showing that bronchoalveolar lavage (BAL) cells from infected animals on 13 dpi were able to kill the NBL, even in the absence of specific antibodies [51]. It can then be speculated that cells have surface effector Igs participating in the ADCC mechanism. The binding of these Igs to the cell surface might be attributed to an upregulation of FcRs triggered by the infection. This phenomenon has previously been described in schistosomiasis by Dombrowicz et al. [52]. Falduto et al. [21] have shown that, as the infection progresses, there is an increase of the percentage of lung leucocytes that express FcεRI, although whether these cells present a greater amount of surface IgE bound to such receptors was not demonstrated. Therefore, whether the leucocytes used in this study are already activated and coated with specific surface antibodies remains to be determined.

## Conclusions

This study provides important data characterizing the immune response triggered in the lamina propria during *T. spiralis* infection. We showed that not only an effector mechanism is directed toward the AW but also the NBL as a cytotoxicity activity was observed against NBL by lamina propria cell suspensions.

## Data Availability

Data supporting the conclusions of this article are provided within the article. The data sets used and/or analysed in the present study are available from the corresponding author upon reasonable request.

## Abbreviations

ADCC: antibody-dependent cell cytotoxicity
ATB: antibiotics
AW: adult worms
AW-ESP: AW excretory-secretory products
BSA: bovine seroalbumin
CCL: chemokine (C-C motif) ligand
CD: cluster of differentiation
dpi: day(s) post-infection
ELISA: enzyme-linked immunosorbent assay
ELISPOT: enzyme-linked immunospot assay
FcεRI: high-affinity receptor for immunoglobulin E
FCS: foetal calf serum
IFNγ: interferon gamma
IIF: indirect immunofluorescence
IL: interleukin
Ig: immunoglobulin
ML: muscle larvae
ML-ESP: ML excretory-secretory products
NBL: newborn larvae
NIRS: non-infected rat sera
OD: optical density (absorbance)
PBS: phosphate-buffered saline
PBST: phosphate-buffered saline plus Tween
RCS: reference cytotoxic serum
SC: secreting cells
SD: standard deviation
TCR: T cell receptor
Th2: type 2 T helper cell (CD4^+^)
TNF-α: tumor necrosis factor alpha
VCU: villus-crypts units
